# CIPHER: a flexible and extensive workflow platform for integrative next-generation sequencing data analysis and genomic regulatory element prediction

**DOI:** 10.1186/s12859-017-1770-1

**Published:** 2017-08-08

**Authors:** Carlos Guzman, Iván D’Orso

**Affiliations:** 1 Department of Microbiology, The University of Texas Southwestern Medical Center, Dallas, TX 75390 USA; 2 Present address: Bioinformatics and Systems Biology Graduate Program, University of California, La Jolla, San Diego, CA 92093 USA

**Keywords:** ChIP-seq, MNase-seq, RNA-seq, DNase-seq, GRO-seq, ATAC-seq, Enhancers, Prediction, Next-generation sequencing, Workflow, Pipeline, Transcription, Gene regulation, Chromatin states, Machine-learning

## Abstract

**Background:**

Next-generation sequencing (NGS) approaches are commonly used to identify key regulatory networks that drive transcriptional programs. Although these technologies are frequently used in biological studies, NGS data analysis remains a challenging, time-consuming, and often irreproducible process. Therefore, there is a need for a comprehensive and flexible workflow platform that can accelerate data processing and analysis so more time can be spent on functional studies.

**Results:**

We have developed an integrative, stand-alone workflow platform, named CIPHER, for the systematic analysis of several commonly used NGS datasets including ChIP-seq, RNA-seq, MNase-seq, DNase-seq, GRO-seq, and ATAC-seq data. CIPHER implements various open source software packages, in-house scripts, and Docker containers to analyze and process single-ended and pair-ended datasets. CIPHER’s pipelines conduct extensive quality and contamination control checks, as well as comprehensive downstream analysis. A typical CIPHER workflow includes: (1) raw sequence evaluation, (2) read trimming and adapter removal, (3) read mapping and quality filtering, (4) visualization track generation, and (5) extensive quality control assessment. Furthermore, CIPHER conducts downstream analysis such as: narrow and broad peak calling, peak annotation, and motif identification for ChIP-seq, differential gene expression analysis for RNA-seq, nucleosome positioning for MNase-seq, DNase hypersensitive site mapping, site annotation and motif identification for DNase-seq, analysis of nascent transcription from Global-Run On (GRO-seq) data, and characterization of chromatin accessibility from ATAC-seq datasets. In addition, CIPHER contains an “analysis” mode that completes complex bioinformatics tasks such as enhancer discovery and provides functions to integrate various datasets together.

**Conclusions:**

Using public and simulated data, we demonstrate that CIPHER is an efficient and comprehensive workflow platform that can analyze several NGS datasets commonly used in genome biology studies. Additionally, CIPHER’s integrative “analysis” mode allows researchers to elicit important biological information from the combined dataset analysis.

**Electronic supplementary material:**

The online version of this article (doi:10.1186/s12859-017-1770-1) contains supplementary material, which is available to authorized users.

## Background

**Fig. 1:**
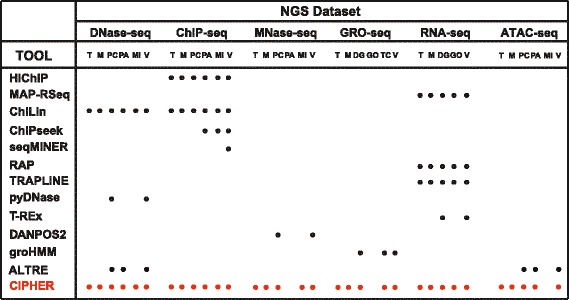
Table of several available workflows for processing sequencing data and their capabilities in comparison to CIPHER. T, Trimming; M, Mapping; PC, Peak Calling; PA, Peak Annotation; MI, Motif Identification; V, Visualization; DG, Differential Gene Expression; GO, Gene Ontology; TC, Transcript Calling

Understanding the precise regulation of transcriptional programs in human health and disease requires the accurate identification and characterization of genomic regulatory networks. Next-generation sequencing (NGS) technologies are powerful, and widely applied tools to map the in vivo genome-wide location of transcription factors (TFs), histone modifications, nascent transcription, nucleosome positioning, and chromatin accessibility features that make up these regulatory networks. Although NGS technologies can be used in diverse ways to investigate numerous aspects of genome biology, reaching sound biological conclusions requires the exhaustive analysis of these datasets to recognize and account for many potential biases [1] including abnormal fragment size distribution due to sonication, bias in enzyme digestion in MNase and DNase samples, PCR amplification bias and duplication, sequencing errors, incorrect software usage, and inaccurate read mappings. These problems, combined with the unprecedented amount of data generated by sequencing platforms, have provided unique opportunities for the development of computational pipelines to automate time-consuming data analysis processes such as ChiLin [2], HiChIP [3], Galaxy [4], MAP-RSeq [5], and bcbionextgen [6], among others (Fig. 1).

Properly implemented pipelines are essential to genome and chromatin biology studies, but often fail to implement the features required to overcome five major challenges: (1) quickly processing large batches of data with minimal user input, (2) remaining highly customizable for different experimental requirements, (3) conducting comprehensive quality control assessments of sequencing datasets to identify potential areas of bias, (4) reducing the issues associated with building, maintaining, and installing multiple pipelines and bioinformatics software, and (5) increasing reproducibility among researchers.

Despite the many computational approaches that already exist to analyze NGS datasets, there are no currently available tools designed to tackle all five challenges simultaneously. ChiLin, HiChIP, bcbio-nextgen, and MAP-RSeq offer powerful command-line data analysis pipelines, but are limited to chromatin immunoprecipitation (ChIP) coupled with sequencing (ChIP-seq) and whole transcriptome sequencing (RNA-seq) studies. Galaxy, an open, web-based platform for data analysis [4], offers an impressive number of bioinformatics tools and workflows that can be used to process various NGS datasets, but severely limits the size and number of files that can be processed at once.

To overcome these previous obstacles, we devised CIPHER, an integrated workflow platform that automates the processing and analysis of several commonly used NGS datasets including ChIP-seq, RNA-seq, Global Run On sequencing (GRO-seq) [7], micrococcal nuclease footprint sequencing (MNase-seq) [8], DNase hypersensitivity sequencing (DNase-seq) [9], and transposase-accessible chromatin using sequencing ATAC-seq [10] datasets. In addition, CIPHER also provides an easy-to-use “analysis” mode that accomplishes complex bioinformatics tasks such as enhancer prediction using a random forest-based machine-learning model and provides functions to integrate various NGS datasets together. By combining Nextflow [11, 12] - a powerful workflow language based on the Unix pipe concept, Docker [13] - a container-based virtualization technology, open source software and custom scripts, we provide a robust, and powerful toolkit that simplifies NGS data analysis and provides a significant improvement over currently available pipelines in terms of flexibility, speed and ease of use.

CIPHER manages to overcome the five previously mentioned obstacles by: (1) parallelizing all the steps in a typical pipeline therefore taking full advantage of a desktop’s or cluster’s available RAM and CPUs, (2) providing command line flags to alter the majority of parameters at each step, (3) incorporating extensive quality control software and providing detailed QC reports specific to each pipeline, (4) combining pipelines for several of the most commonly used NGS techniques into a single, standalone tool, and (5) using a lightweight Docker containers to package all the required software dependencies to run CIPHER into a standardized environment.

In this report, we demonstrate that CIPHER is a fast, reproducible, and flexible tool that accurately processes and integrates NGS datasets by recreating the results of two published studies, and comparing CIPHER’s speed and ease of use to two other ChIP-seq and RNA-seq pipelines. We further validate CIPHER’s built-in random-forest based enhancer prediction model by identifying potentially functional enhancers in various human cell lines.

## Implementation

Many previously described NGS workflows are developed using scripting languages such as Python or Perl as a ‘glue’ to parse datasets, and automate the series of commands that make up a processing pipeline. In contrast to these approaches, CIPHER was designed using Nextflow, a specialized, and new workflow language that is built around the Unix pipe concept [11]. By using Nextflow as the underlying language for the CIPHER platform, we gain access to several useful features, including automatic parallelization, Docker and GitHub support, the capacity to run locally on a desktop or on a cluster, and the ability to seamlessly integrate custom scripts in a variety of programming languages.

CIPHER can be run with default settings by specifying the “--mode”, “--config”, “--readLen”, “--lib”, “--fasta”, and “--gtf” flags. The “--mode” flag indicates the type of NGS pipeline you wish to run from the currently available workflows (e.g. “--mode chip” for ChIP-seq analysis), while the “--config”, “--readLen” and “--lib” flags provide information regarding file locations, read length and type of sequencing (e.g. single-ended or pair-ended), respectively, so that the pipeline runs the appropriate processes. Finally, the “--fasta” and “--gtf” flags indicate reference annotation information that is required for mapping and downstream exploration such as differential gene expression (DGE) analysis. In the case that the user is not familiar with reference FASTA and GTF files or where to acquire them, providing the “--download_data” flag will automatically download the appropriate Ensembl/Gencode reference files for a specified organism, if it exists (e.g. “--download_data hg19” will download Gencode fasta and gtf files for the hg19 human genome).

In addition, there are various other flags that can be set to customize the analysis further. More information regarding these flags can be found by setting the “--help” flag or by visiting CIPHER’s online documentation (available at: cipher.readthedocs.io). By default, CIPHER will output processed files into a “./report” directory (which can be changed by specifying the “--outdir” flag). The output includes various files and is largely dependent on the pipeline mode specified, but in general provides quality control reports in pdf or html format, gzipped fastq files of raw sequences after trimming and adapter removal, sorted BAM files of mapped and unmapped alignments along with various files that contain detailed statistics regarding the number of unique, multimapped, and low-quality reads, as well as normalized track files in bigWig format for visualization. Further pipeline dependent downstream analysis such as narrow and broad peak calls for ChIP-seq, differential gene expression lists for RNA-seq, nucleosome positions and DNase hypersensitive sites (DHS) for MNase-seq and DNase-seq respectively, unannotated transcription units for GRO-seq, and chromatin accessible sites for ATAC-seq are also output. Notably, unlike other published pipelines (Fig. 1), CIPHER is the first platform that merges multiple workflows and complicated bioinformatics tools into a single, easy to use, parallelized, and scalable toolkit, removing the obstacles that arise from finding, building, maintaining, and updating multiple workflows. This approach can be applied to data generated through both pair-ended and single-ended sequencing to map genomic elements and regulatory features in diverse organisms.

**Fig. 2:**
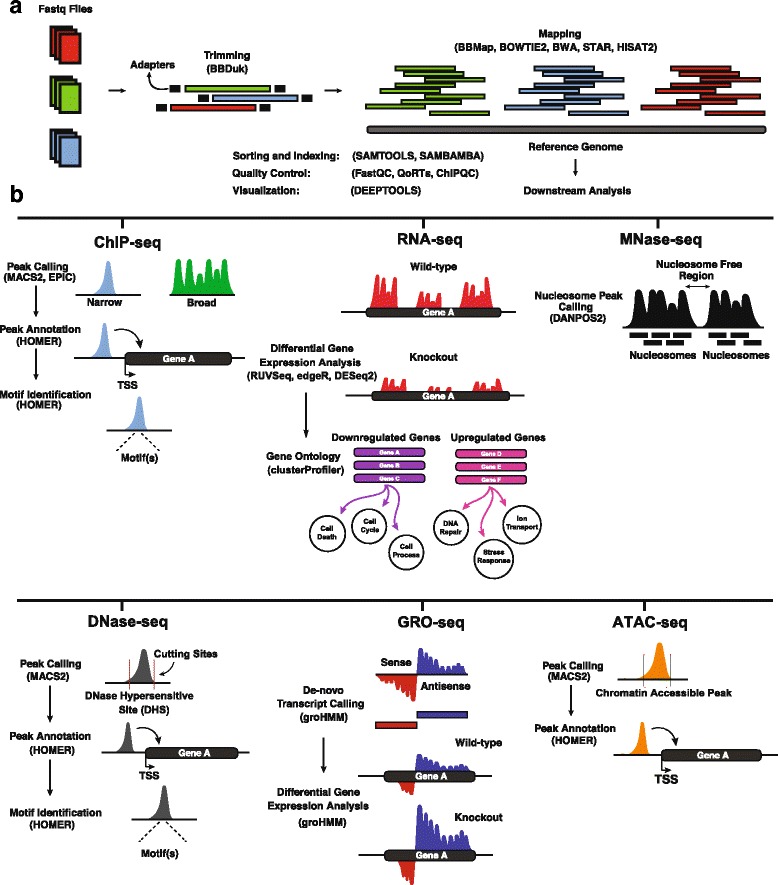
Brief visual representation of CIPHER’s two stage workflows. **a** Fastq files are trimmed of adapters and low quality reads using BBDuk, and then mapped to the reference genome using user’s preferred aligner. **b** Mapped reads are then run through a downstream analysis pipeline that reveals biological functions and is dependent on the type of dataset input. See text for complete details

CIPHER’s pipelines conduct extensive quality and contamination control checks, as well as comprehensive downstream analysis (Fig. 2). A typical CIPHER workflow can be split into two major stages: a fastq sequence filtering, adapter trimming, and read mapping stage, and a downstream analysis stage. During the ‘sequence filtering, trimming, and mapping’ stage, raw sequences are trimmed of adapters and low-quality reads using BBDuk [14], and are then mapped to a reference genome (Fig. 2a). CIPHER allows the user to choose between three different aligners for non-splice aware datasets: BBMap [14], the Burrow-Wheeler Aligner (BWA) [15] and Bowtie2 [16], and three different aligners for splice aware datasets: BBMap [14], STAR [17], and HISAT2 [18] via the “--aligner” flag. After mapping, the ‘downstream analysis’ stage consists of running the samples through various steps to extract biological information including peak calling for narrow (MACS2) and broad binding domains (EPIC), peak annotation and motif identification (HOMER) [19] for ChIP-seq; DGE analysis for RNA-seq (RUVSeq, edgeR, and DESeq2); analysis of nascent transcription from GRO-seq (groHMM); nucleosome positioning for MNase-seq (DANPOS2); positioning/strength of DHS (MACS2), site annotation (HOMER), and motif identification (HOMER) for DNase-seq; and chromatin accessibility peak calling (MACS2), and annotation for ATAC-seq (HOMER) (Fig. 2b). Overall, CIPHER ensures comprehensive, reproducible, customizable, and accurate automated NGS dataset processing (see below).

### Trimming

Trimming adapter sequences is a common pre-processing step during NGS data analysis, as adapter contamination can often disturb downstream examination. Many tools exist for the removal of adapters such as Trimmomatic [20], cutadapt [21], Trim Galore [22], and BBDuk [14]. CIPHER implements BBDuk, which is an extremely fast, scalable, and memory-efficient decontamination tool to remove Illumina, Nextera, and small RNA adapters from raw sequencing data. By default, CIPHER will also filter out low-quality (default: mapq <20) and short-length (default: length < 10) reads as this has been shown to increase the quality and reliability of downstream analysis [23]. Additional adapter sequences can be added manually to an “adapters.fa” file located in CIPHER’s “bin” directory.

### Mapping

Mapping or alignment, while generally being the most computationally intensive part of any pipeline, is also a crucial and often confusing pre-processing step. Low mapping efficiencies can be caused by numerous issues including adapter or organismal contamination, poor sequence quality, high-levels of ribosomal RNA content, poor library-preparation quality, and/or inappropriate parameter use, which can often lead to incorrect or inefficient downstream analysis.

While several mapping software packages have been developed to map reads to a reference genome, they are typically designed to address a specific type of data or sequencing technology. For example, ChIP-seq data makes use of splice-unaware aligners such as BWA [15], Bowtie2 [16], and BBMap [14] while RNA-seq data requires a splice-aware aligner to avoid introducing long gaps in the mapping of a read due to intronic regions, and thus leading to false mappings. Several splice-aware aligners exist including BBMap [14], STAR [17], and HISAT2 [18].

Notably, CIPHER integrates pipelines that require splice-aware (RNA-seq) and splice-unaware mappers (ChIP-seq, MNase-seq, DNase-seq, ATAC-seq and GRO-seq), and supports both single-ended and pair-ended sequencing datasets. Thus, to appeal a broader audience, CIPHER allows the user to choose from five different aligners (BBMap, BWA, Bowtie2, HISAT2, and STAR) to fit any experimental condition and dataset.

By default, CIPHER will map reads to a reference genome using BBMap, a fast short-read aligner for both DNA and RNA-seq datasets, that is capable of mapping very large genomes containing millions of scaffolds with very high-sensitivity and error tolerance. Because read alignment often requires a large amount of flexibility for specific datasets (e.g. removing the first 5 nucleotides from the 5′ end of a read), CIPHER enables the user to set all of an aligner’s parameters at the top level.

### Quality control

To ensure properly sound biological conclusions, it is crucial that the user accurately and thoroughly evaluates the quality of their sequencing datasets. To this end, CIPHER incorporates a number of quality control tools to identify potential biases, contaminations, and errors in NGS datasets.

All pipelines integrate FastQC [24], an open source module that is used to analyze raw sequencing datasets for any abnormalities such as high duplication levels or adapter contamination, as well as low-quality and short-length reads. ChIP-seq, DNase-seq, ATAC-seq, and MNase-seq datasets are run through ChIPQC [25], an R package that automatically computes several quality control metrics including the total number of reads in each BAM file per sample, mapping statistics (e.g. number of successfully mapped reads, number of mapped reads with a quality score less than *N*, multimappers), estimated fragment length by calculating cross-coverage score, and the percentage of reads that overlap called peaks (known as FRIP) when possible.

Fingerprint plots that predict enrichment of ChIP-seq datasets are generated to judge how well a ChIP experiment worked using deeptool’s [26] “plotFingerprint” function. For RNA-seq datasets, QoRTs [27] is used to detect and identify various errors, biases, and artifacts produced by single-ended and pair-ended sequencing. Furthermore, RNA-seq data is run through Preseq [28] to predict the yield of distinct reads from a genomic library after an initial sequencing experiment. These predictions can be used to examine the value of further sequencing, optimize sequencing depth, or screen multiple libraries to avoid low complexity samples by estimating the number of redundant reads from a given sequencing depth.

MultiQC [29] is used to aggregate the results from various quality control files into a single, easy to read HTML report, summarizing the output from numerous bioinformatics tools such as FastQC so that potential problems can be detected more easily and output can be parsed by the user quickly.

### Peak calling

For our purposes, peak calling refers to the identification of TF and histone binding domains, nucleosome positions, DHS, and chromatin accessible sites (Fig. 2). There are two major types of ChIP-seq binding profiles: narrow and broad binding (Fig. 2b). Narrow peak calls are typically accomplished by identifying locations with an extreme number of reads as compared to an input, while broad peak calls are more concerned with determining the edges or boundaries of these diffuse peaks. Because the mechanisms of discovery for these binding domains are very different, CIPHER integrates, at difference to other pipelines, two different software algorithms. For narrow binding profiles, MACS2 [30] is used to identify candidate regions by using a dynamic Poisson distribution to capture background levels, and scans the genome for enriched overlapping regions which are then merged into peaks. For broad binding profiles, EPIC [31], a fast, parallel and memory efficient implementation of the SICER [32] algorithm, is used. EPIC improves on the original SICER by taking advantage of the advances in Python data science libraries, such as the Pandas module, to improve the algorithm’s proficiency and handle the large amounts of data that the original software is unable to.

By default, CIPHER will estimate the fragment length for each sample using the SPP R package [33], and bypass MACS2’s shifting model using the “--nomodel” flag. Each read is extended in a 5′ - > 3′ direction using the “--extsize” flag set to the estimated fragment size. CIPHER also will use false discovery rate (FDR) values as a cutoff to call significant regions (default: “--qvalue 0.01”). Narrow peaks are called for samples with a control (e.g. Input) or without. All duplicate tags are kept (that is all tags in the same orientation and strand) using the “--keep-dup all” flag. Broad peaks are only called for samples with a control. Similarly to MACS2, reads are extended to estimated fragment size. EPIC pools all windows with sequencing reads together and estimates a composite score, allowing very long stretches of broad signal (such as some chromatin domains) to be detected. By default, CIPHER will scan the genome by separating them into 200-bp windows. Enriched broad regions are estimated and an FDR score is calculated for each region, those that fall beneath the provided cutoff (default: 0.01) are not reported.

Nucleosome positions are determined using the DANPOS2 software suite [34] (Fig. 2b). DANPOS2 is a toolkit for the statistical analysis of nucleosome positioning, including changes in location, fuzziness, and occupancy. The “dpos” function from the DANPOS2 toolkit is used to identify nucleosome positions from MNase-seq datasets. Fragment size is automatically calculated by CIPHER as previously mentioned, and several flags can be set to specify read density cutoffs, window size, merge distance, wiggle step size, and wig smoothing size to accommodate different datasets, as explained in detail in the user manual available at cipher.readthedocs.io.

DHS characterize chromatin accessible regions in the genome where TFs can bind (Fig. 2b). While several DNase-seq specific peak callers such as F-seq [35] have been developed, studies have also shown that MACS2 can be used to accurately predict DNase hypersensitive positions [36]. Thus, to limit the number of dependencies, CIPHER uses MACS2 to identify chromatin accessible regions from DNase-seq data. DHS are predicted in a similar manner to narrow ChIP-seq binding sites, except a combination of the “--extsize” and “--shift” flags are used to shift the ‘cutting’ ends (e.g. sites where DNase cuts the DNA) and then reads are extended into fragments. By default, reads are shifted by calculating “-1 * one-half the estimated fragment size” as indicated in the MACS2 manual.

A similar approach to the identification of chromatin accessible sites from ATAC-seq data is used. CIPHER takes advantage of MACS2 flexible algorithm to call peaks in a similar manner to DHS. However, “--extsize” of 73 and “--shift” of −37 is used since the DNA wrapped around a nucleosome is about 147-bp in length.

### Visualization

To visualize binding site, gene expression, chromatin accessibility, and gene annotation information, various visualization tracks (e.g. bedGraphs and bigWigs) are produced. Deeptools [26] is used to generate bigWig’s for every workflow. All tracks are normalized by reads per genomic content (RPGC), which reports read coverage normalized to 1X sequencing depth. Sequencing depth is defined as the total number of mapped reads times the fragment length divided by the effective genome size (EGS). CIPHER automatically calculates EGS using EPIC’s “epic-effective.sh” script. ChIP-seq, MNase-seq, DNase-seq, and ATAC-seq datasets have their reads extended to their estimated fragment size, while RNA-seq and GRO-seq datasets do not. CIPHER outputs sense and anti-sense bigWigs for RNA-seq and GRO-seq datasets indicative of sense and anti-sense transcription. Furthermore, CIPHER outputs RPM-normalized bedGraph files via MACS2 that can be used in some “analysis” mode functions.

### Differential gene expression

DGE analysis generally refers to the up- or down-regulation of transcripts produced by a cell in response to or because of an aggravation (e.g. knock-out of a gene/genomic domain or knock-down of a certain factor). CIPHER’s DGE pipeline is straightforward and includes basic mapping, quantification, and DGE analysis steps. As previously described, mapping is completed by the user’s choice of aligner, while quantification is accomplished by the featureCounts module [37] of the Subread suite. Featurecounts is a fast, general purpose read summarization program that counts mapped reads for genomic features such as genes. Actual DGE analysis is completed by both the edgeR [38] and DESeq2 [39] packages from Bioconductor, as they are the most commonly used DGE packages in publications.

### Enhancer prediction

**Fig. 3:**
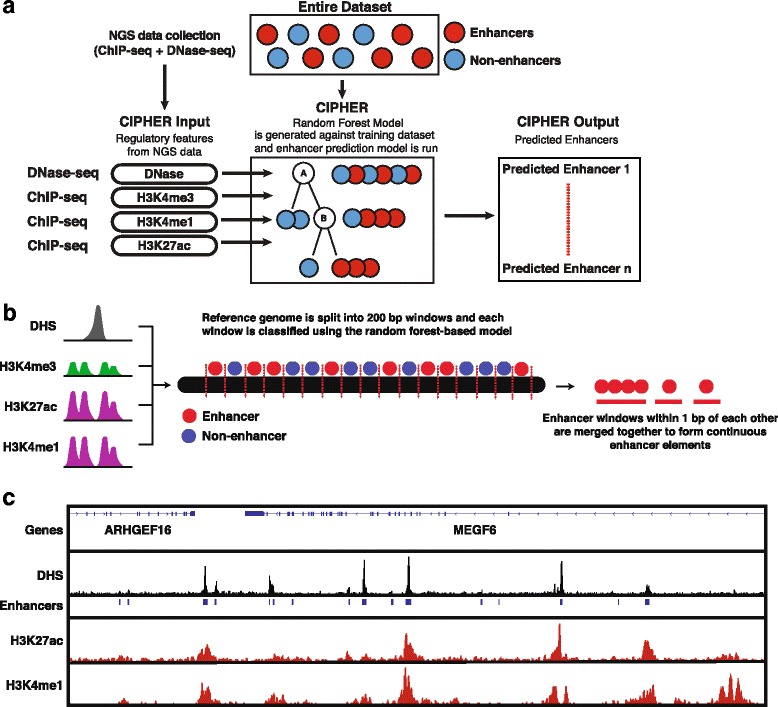
Outline of the random forest machine learning process for enhancer prediction by CIPHER. **a** Enhancer elements can be identified de novo in a preferred cell line by using select histone modification and chromatin accessibility data and inputting it into CIPHER, which will then output a list of predicted enhancer elements by applying the model to the cell line. Genomic features (histone modification and chromatin accessibility data) are calculated for defined enhancers obtained from the ENCODE project. Non-enhancer elements are promoter regions −/+ 1 Kb from the TSS of all known genes. A subset of all enhancer and non-enhancer elements is split into two groups: (1) a testing and (2) a training dataset. The training dataset is used to generate the machine-learning model where decision trees are generated until the model can effectively separate enhancers from non-enhancers. The testing dataset is used to validate the model, and a confusion matrix is used to calculate the accuracy of the model. **b** Enhancer identification workflow. DNase chromatin accessibility (DHS) and chromatin signatures (H3K4me1, H3K4me3, and H3K27ac) are used as input data. CIPHER splits the reference genome into 200-bp windows and then applies its random forest-based machine learning model to each reference window to classify each window as an ‘enhancer’ or ‘non-enhancer’. Enhancer windows are then merged so that windows within 1 bp of each other form a single continuous enhancer element. **c** Genome browser tracks of DHS and enhancer signature markers (H3K27ac and H3K4me1) alongside the position of the predicted enhancer elements (*blue blocks*) output by CIPHER’s machine learning model

Enhancers are short DNA sequences that act as TF binding hubs, and function in a spatio-independent manner to fine-tune promoter activity at distances ranging hundreds of bases to megabases. To predict enhancers, we developed and applied a random-forest tree (RFT) machine-learning model that combines chromatin accessibility (DNase-seq) and chromatin signature datasets obtained from ChIP-seq (H3K4me1, H3K27ac, and H3K4me3) (Fig. 3a). The RFT model (implemented in R (version 3.3.1) using the *randomForest* package) was constructed using the classical concept of binary classification trees, with each feature being the average coverage signal of a marker within a set distance along a genomic element. CIPHER takes RPM-normalized bedgraph files of DNase-seq and ChIP-seq as input to build the RFT model.

RFT model construction underwent two stages: training and testing. In the ‘training’ stage, a forest is constructed using two classes of genomic elements (one class containing a previously determined set of enhancer elements from the Encyclopedia of DNA Elements (ENCODE) project [40] and a second class with an equal number of promoter regions (−1/+1 Kb from the transcription start site (TSS)). In the ‘testing’ stage, a third of the classes and their classifications that are not used for training are selected to test the accuracy of the generated RFT-model. The accuracy of the model was tested using a confusion matrix from the *caret* package in R. Notably, CIPHER’s enhancer prediction-model accuracy achieved slightly above 93%, which means that the majority of ‘true’ enhancers were identified during our ‘testing’ stage, indicating reliably efficient enhancer identification functionality.

The provided reference genome is split into 200-bp bins, and the enhancer prediction model categorizes each window into “enhancer” or “non-enhancer” bins (Fig. 3b). Bins that are within 1-bp of each other are further merged to form a single continuous region. To account for false-positive enhancer predictions, we set a strict cut-off using DHS peaks whereby a DNase associated peak must overlap the predicted enhancer by at least a single bp (q < 0.01, MACS2) to be considered a ‘validated’ enhancer and output as a result (Fig. 3c).

### Analysis mode

CIPHER’s “analysis” mode was created to take advantage of CIPHER’s broad NGS data processing workflows. In “analysis” mode, CIPHER can run several functions that integrate various input files and combines them to answer a more specific or typically more complex biological question. Currently, CIPHER contains two main analysis functions. We have already touched on CIPHER’s enhancer prediction functionality, but “analysis” mode also contains a “geneExpressionNearPeaks” function that calculates fragments per kilobase per million mapped reads (FPKM) and transcripts per kilobase per million mapped reads (TPM) normalized expression values of genes near an input list (e.g. list of peaks or enhancers). This is accomplished by taking the Stringtie [41] output file from an RNA-seq experiment and a list of MACS2/EPIC called peaks from ChIP-seq and identifying the nearest gene to each peak and then merging the information. By taking advantage of this “analysis” mode we hope CIPHER provides a much more integrative tool-kit that expands beyond simple data processing.

## Results

To validate CIPHER’s potential in NGS data analysis, we used data from the Gene Expression Omnibus repository (GEO) to re-create two previously published studies: a ChIP-seq study from McNamara et al. [42] and a GRO-seq study from Liu et al. [43]. Furthermore, we briefly compared CIPHER’s speed, and ease of use to alternative pipelines such as HiChIP and MAP-RSeq. Next, we used real and simulated data to evaluate and describe how to compare the performance of various adapter decontamination tools (BBDuk, Cutadapt and Trimmomatic) and DNA mappers (BBMap, BWA, and Bowtie2) using ENCODE datasets. Finally, we confirm CIPHER’s enhancer-prediction model by calling enhancers in three human cell lines. Performance tests were run on a 32 core, dual-core Intel Xeon E5 with 128GB RAM WhisperStation.

### Validating CIPHER’s pre-processing abilities and accuracy

To determine if CIPHER’s workflows are appropriate for typical NGS studies, we downloaded the raw data from two studies [42, 43] and ran them through CIPHER to attempt reproduce their conclusions.

The first study by McNamara et al. consisted of several ChIP-seq datasets, and provided evidence that KAP1, also known as TRIM28, acts as a scaffold to recruit the 7SK snRNP complex to gene promoters to facilitate productive transcription elongation in response to stimulation. Their bioinformatics analysis showed that 70% of all genes in the human genome containing a form of RNA polymerase II (Pol II) that is paused at promoter-proximal regions (defined as −250/+1000 from the known TSS), also contained the KAP1-7SK snRNP complex as revealed by co-occupancy of KAP1 and three subunits of the 7SK snRNP complex (HEXIM, LARP7, and CDK9). The same authors also published a thorough methods paper that provided a detailed experimental description and analysis of ChIP-seq datasets [44], in which mapping to the UCSC hg19 genome was completed by Bowtie [45] and peak calling was accomplished by MACS2. The study led to the identification of 14,203 target genes in the human genome containing this regulatory complex.

To determine whether we could reproduce McNamara et al.’s results using CIPHER, we processed six of their ChIP-seq datasets (Pol II, KAP1, HEXIM, LARP7, CDK9 and Input). Given that CIPHER does not include a Bowtie aligner, we used the more recent Bowtie2 aligner (“--aligner bowtie2” flag in CIPHER) but all other settings were left as default. CIPHER processed all ChIP-seq datasets (~30 million reads per dataset) in 7 h and 23 min. We then identified ~26,000 promoter-proximal regions (defined as in the original manuscript (−250/+1000-bp from known TSS)) and conducted co-occupancy analysis of called peaks. Our analysis revealed 14,397 KAP1-7SK snRNP target genes as opposed to the original manuscript’s 14,203 (Table 1) (Additional file 1: Table S1).

**Table 1:** Comparing CIPHER’s output with original publication results

	CIPHER	Original Publication
KAP1-7SK snRNP Target Genes	14,397	14,203
BRD4-KD DGE Genes	2528	2549

A table of re-created results from original publication data using CIPHER

The second study by Liu et al. consisted of several GRO-seq datasets to explore the role of two human factors (JMJD6 and BRD4) on the activation of the Pol II paused form in a process called ‘Pol II pause release’ [43]. The study is quite elaborate, but does include a number of DEG that are central to the paper for either the JMJD6 (386 down-regulated; 1722 up-regulated) and/or BRD4 (744 down-regulated; 1805 up-regulated) complex subunits. According to their methods, all reads were aligned to the hg19 RefSeq genome by Bowtie2, and feature counting was completed by HOMER. EdgeR was used to compute actual DEG at a FDR of <0.001.

We decided to reproduce one important section of this previous study by processing six GRO-seq datasets: 2 non-target (NT) replicates, and 4 Brd4 knockdown (KD) replicates. As previously done, we left all settings at default except for altering the “--aligner” flag to use Bowtie2. CIPHER processed all six GRO-seq datasets (~50 million reads per dataset) in approximately 10 h. DGE analysis of NT versus BRD4 KD resulted in 2528 differentially expressed genes at an FDR < 0.001 (Table 1). We then overlapped both gene sets and found that CIPHER called 98% of the same genes as reported in the Liu et al. study, providing compelling evidence that CIPHER can be used, even with default settings, to accurately process and analyze various NGS datasets.

### Ease-of-use and speed comparisons of CIPHER versus alternative pipelines

The adoption of new software is largely dependent on proper documentation, and how easy the new software is to install and use when compared to other alternatives. Here we briefly examined and compared the speed and ease-of-use of CIPHER versus two other pipelines (HiChIP for ChIP-seq and MAP-RSeq for RNA-seq).

We first downloaded and installed both HiChIP and MAP-RSeq standalone versions. While both pipelines provided virtual machines (VM) that already came packaged with all the necessary software and dependencies, we found that these VMs were clunky, slow and only really meant to demonstrate the pipeline for testing purposes. While both pipelines provided detailed instructions on how to manually install all the software and dependencies that were required, users who are unfamiliar with bash or Unix commands would have significant trouble installing them. Furthermore, both pipelines provided a version of their pipelines that could only be run exclusively on a SGE cluster, greatly limiting their use.

In comparison, CIPHER only requires the manual installation of Nextflow and Docker, greatly reducing the number of obstacles a new user may encounter during their setup. By default, CIPHER will automatically fetch Docker containers that hold all the required software and dependencies that are needed to run the pipeline, without the slow-down that comes with a typical VM. In cases where the user does not or cannot use/install Docker, we have provided detailed instructions on how to download all the software required to run CIPHER using the Anaconda package manager in our documentation (cipher.readthedocs.io). Importantly, CIPHER can be easily run on several cluster services including SGE, SLURM, LSF, PBS/Torque, NQSII, HTCondor, DRMAA, DNAnexus, Ignite, and Kubernetes without altering the original script, thus vastly increasing the flexibility and usage of our workflow platform.

We next compared the difficulties in running each of the pipelines on several ChIP-seq and RNA-seq samples. We discovered that HiChIP required three configuration files and MAP-RSeq required four configuration files that need to be modified and completed before the workflow can be run, leading to an extremely tedious pipeline setup process. In contrast, CIPHER only requires the creation of a single configuration file that contains the merge ID, sample ID, path(s) to fastq(s), control ID, and marker ID for each sample vastly reducing the time and complexity of the initial startup.

Finally, we ran each of the pipelines on single and multiple in-house datasets to test their speed. For ChIP-seq we first ran a single sample and its associated input (~30 million reads each) and then conducted another run that included five samples and their associated input (~30 million reads each). We found that for the single sample dataset, HiChIP took approximately 2X longer than CIPHER (~8 h versus ~4 h, respectively). However, the difference in run time became vastly more noticeable when the pipelines were run on multiple datasets (6 samples), in which HiChIP took approximately 4X longer to finish than CIPHER (~30 h versus ~8 h, respectively).

Fairly similar results were obtained with the MAP-RSeq pipeline, where processing a single RNA-seq sample (~50 million reads; no DGE analysis) took approximately 1.5X as long using MAP-RSeq than CIPHER (~8 h versus ~6 h), while processing 18 samples (~50 million each; no DGE analysis) took approximately 15X as long using the MAP-RSeq pipeline (~126 h, run was stopped after 72 h versus ~8 h). These speed differences are likely the result of CIPHER’s innate ability to process a large number of datasets in parallel, while both HiChIP and MAP-RSeq have to process datasets serially (e.g. one at a time). Together this demonstrates the ease-of-use and speed of CIPHER compared with other pipelines.

### Adapter decontamination tool performance tests on down-sampled ENCODE datasets

We downloaded ChIP-seq (H3K4me1) from the ENCODE project in human colonic cancer cells (HCT116) to obtain real quality distributions. We then down-sampled the original fastq files into three different datasets containing 1 M, 5 M and 10 M reads using BBMap’s “reformat.sh” script. Using a dataset of 25 Ilumina TruSeq adapters we randomly added adapters to the reads using the “addadapters.sh” script from the BBMap suite with “qout = 33” and “right” flags set, to ensure that adapters will be 3′-type adapters. This ensures that adapters will be added at a random location from 0 to 149, and possibly run off the 3′ end of the read, but the read length always stays at 150. If the adapter finishes before the end of the read, random bases are used to fill in the rest. Using this approach, about 50% of all reads get adapters. Once the adapter is added, each of the adapter nucleotides is possibly changed into a new nucleotide, with a probability from the read’s quality score for that nucleotide to simulate sequencing error.

Speed tests were conducted using the “time” Unix command for 1 M, 5 M and 10 M reads and averaging the completion times over 3 runs. Accuracy was estimated by replacing each read’s original name with a synthetic name indicating the read’s original length and length after trimming. For example, “@0_150_15” means that the read was originally 150 bp long and 15 bp after trimming because an adapter was added at position 15 (0-based). This allows BBMap’s “addadapters.sh” script (with the “grade” flag set) to quantify both the number of bases correctly and incorrectly removed, as well as the percentage of true-positive adapter sequences remaining and non-adapters removed.

**Fig. 4:**
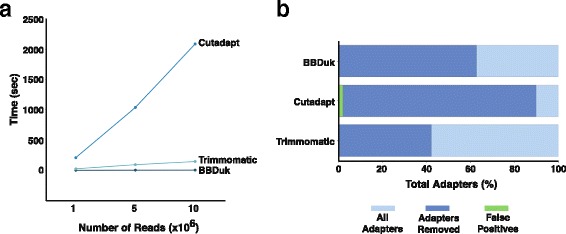
Performance tests for various adapter decontamination tools. **a** A line graph indicating the number of reads processed on the x-axis and the time in seconds the tool used to process that number of reads on the y-axis. **b** A barplot indicating the percentage of total adapters (*light blue*), adapters removed (*dark blue*), and incorrectly removed adapters (false positives, *green*) for each tool tested

Performance tests showed that BBDuk outperforms the speed category by a large margin, with Trimmomatic not far behind, and Cutadapt being extremely slow (Fig. 4a) while accuracy tests revealed that Cutadapt removes more correct adapters, with BBDuk following closely behind and Trimmomatic at the end (Fig. 4b). However, Cutadapt removed two times more incorrect adapter sequences than other trimmers resulting in a higher amount false-positive adapter trimming (Table 2). Taken together, the combined speed and accuracy of BBDuk, along with its easy to use parameters, and ability to work on single-ended as well as pair-ended sequencing, make it a great choice for read trimming and adapter removal.

**Table 2:** Summary of performance statistics for various trimming tools and number of reads

Tool	Number of Reads	Speed (sec)	Adapters Remaining (%)	False Positives (%)
BBDuk	1 M	1.2		
BBDuk	5 M	2.3	37.2	0.0099
BBDuk	10 M	3.5		
Cutadapt	1 M	208		
Cutadapt	5 M	1037	10.1	2.1059
Cutadapt	10 M	2084		
Trimmomatic	1 M	25		
Trimmomatic	5 M	93	57.7	0.0004
Trimmomatic	10 M	145		

A table of performance tests and statistics between various trimming tools. Each tool was tested on datasets with 1 M, 5 M or 10 M reads. Speed tests were averaged across three replications. Adapters remaining and false positives tests were only conducted on 5 M read datasets, as the difference between 1 M, 5 M, and 10 M datasets was minimal

### Alignment tool performance tests on simulated datasets

**Fig. 5:**
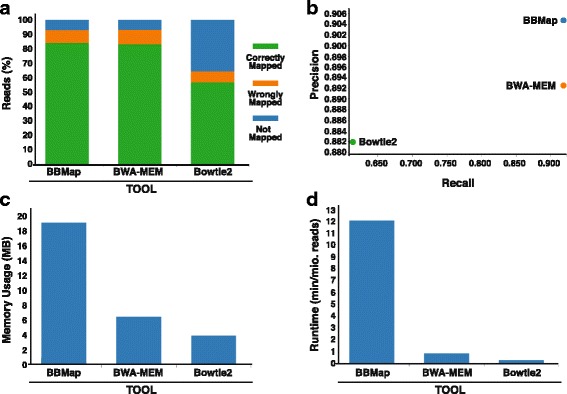
Performance tests for various mapping tools. **a** A barplot indicating the percentage of reads correctly mapped (*green*), wrongly mapped (*orange*), and not mapped (*blue*) for each tool. **b** A scatter plot indicating the precision and recall rates for each tool. Top right indicates best performance overall. **c** A barplot of memory usage in MB for each tool. **d** A barplot of the number of minutes per million reads mapped each tool took

To compare mappers against each other, we generated a dataset using Teaser [46], which is a tool that can be used to analyze the performance of various read mappers on simulated or real world datasets. We simulated a single human Illumina-like read set assuming a genomic SNP frequency of 0.1% and a 0.3% probability for the occurrence of insertions and deletions. Read length for the simulated dataset was set to 100 bp and assumed a sequencing error of 0.6%. To reduce computing times, we had Teaser randomly sample 0.01% of non-overlapping sequences from the genome. The simulated reads were then mapped to the entire UCSC hg38 reference genome and mapping statistics were evaluated (Fig. 5). All mappers were run in Teaser’s default mode with no additional parameters unless otherwise indicated.

Results showed that BBMap and BWA-MEM correctly mapped more simulated reads (83.889% and 82.863%, respectively) than Bowtie2 (56.545%) (Fig. 5a). All three tools mapped ~7–10% of reads incorrectly (defined as reads that mapped to incorrect loci), but Bowtie2 was not able to map 35% of simulated reads to the human genome at all compared to the ~7–10% of unmapped reads for BBMap and BWA-MEM. Teaser also reported the precision (fraction of correctly mapped reads compared to all mapped reads) and the recall rate (fraction of correctly mapped reads if compared to correctly mapped reads and non-mapped reads) for each mapper. Not surprisingly, BBMap achieved the highest precision and recall rating at 90.47% and 92.03% respectively, with BWA-MEM close behind at 89.24% precision and 92.07% recall and Bowtie2 performing significantly worse (88.18% precision and 61.19% recall rating) (Fig. 5b).

BBMap, BWA-MEM and Bowtie2 appear to perform on par in terms of accuracy, performance tests for memory usage and speed indicated that BBMap was slower and used larger amounts of RAM than either of the other two programs (Fig. 5c and d). However, BBMap builds its index on the fly and thus its resulting time is not indicative of its pure mapping speed. In conclusion, we propose that all three mappers perform comparatively well on our simulated dataset, with Bowtie2 showing slightly lower performance test results in several sections. It is important to keep in mind that all aligners can be altered quite significantly to achieve higher sensitivity, and improve mapping results, and in our case, we only tested the mappers using their default settings and levels of stringency. Taken together, CIPHER offers ample alignment/mapping opportunities giving the user a broad spectrum of pipelines to be selected depending on their specific needs and biological questions to be answered.

### Enhancer-identification model validation

Enhancers are short DNA-sequences that can regulate basal gene transcription over distances ranging from a few kilobases to megabases. Enhancers are characterized by the presence of various genomic features including: (1) an accessible chromatin landscape, (2) distinct chromatin signatures, (3) TF binding, and (4) bi-directional transcriptional activity as revealed by the presence of enhancer-derived non-coding RNAs (eRNAs) based on GRO-seq data [47, 48].

Previous studies have shown that it is possible to accurately predict enhancer elements using machine-learning models by combining these various regulatory features [49–53]. However, most of this enhancer-prediction modeling is bundled into software that is highly technical in nature and often requires specialized paid software such as MATLAB to use. To simplify enhancer identification, CIPHER implements a random-forest based classifier similar to the model developed by Bing Ren’s group at UCSD [50] (Fig. 3). Our model predicts transcriptional enhancers based on a combination of chromatin signatures (H3K4me1, H3K27Ac, H3K4me3) and DNase-seq information.

To validate CIPHER’s enhancer prediction functionality, we identified enhancer elements in two cell lines (HCT116 and HeLa). Using ChIP-seq and DNase-seq datasets from the ENCODE project, we generated average coverage profiles for H3K4me1, H3K27ac, H3K4me3, and DNase-seq. These coverages profiles were fed into CIPHER’s “analysis” mode.

**Fig. 6:**
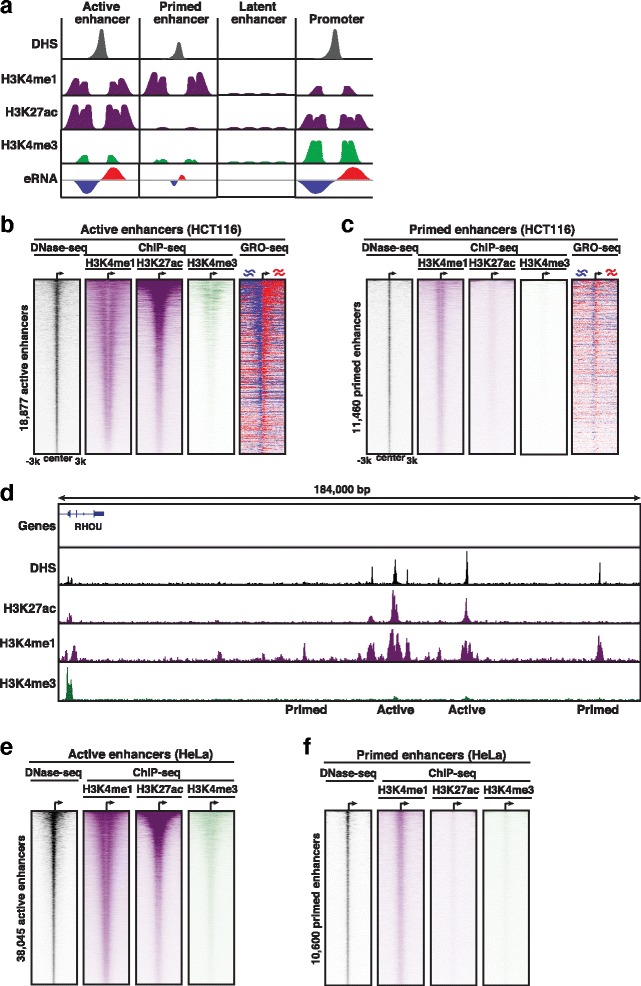
Definition of enhancer states and validation of enhancer-prediction model. **a** Enhancer states and their corresponding chromatin signatures, DNase hypersensitive sites (DHS), and eRNA levels. *Red* and *blue* indicate sense and antisense eRNAs, respectively. **b** Heat maps of DNase, H3K4me1, H3K27ac, H3K4me3, and GRO-seq (*red*: sense eRNAs, *blue*: antisense eRNAs) signal at active enhancers centered on the middle of all enhancers and extended 3 Kb in either direction. **c** Heat maps of DNase, H3K4me1, H3K27ac, H3K4me3, and GRO-seq (*red*: sense eRNAs, *blue*: antisense eRNAs) signal at primed enhancers centered on the middle of all enhancers and extended 3 Kb in either direction. **d** Genome browser view of predicted enhancers and their associated genome features. **e** Heat maps of HeLa active enhancers centered on the middle of all enhancers and extended 3 Kb in either direction. **f** Heat maps of HeLa primed enhancers centered on the middle of all enhancers and extended 3 kb in either direction

Enhancer activity can be inferred from the presence or absence of histone markers. Enhancers are typically marked with high levels of H3K4me1, in contrast to promoters that are marked with higher levels of H3K4me3. More recently, H3K27ac and high eRNA content have been found to distinguish functionally active from primed or latent enhancers [47, 54] (Fig. 6a). Thus, predicted enhancers were further divided into active and primed enhancer ‘states’ based on their H3K27ac levels or lack thereof, respectively. CIPHER predicted 18,877 active and 11,460 primed elements in HCT116 and 38,045 active and 10,600 primed elements that contained the expected DNase-sensitivity pattern (DNase-seq), chromatin signatures (ChIP-seq), and transcriptional activity content (GRO-seq) (Fig. 6b and c).

Chromatin state profiles were evaluated by constructing heatmaps for active and primed enhancers ranked by decreasing levels of chromatin accessibility (Fig. 6b and c). This analysis revealed accessible chromatin at the center of all predicted enhancers as shown by DNase-seq, and chromatin signatures surrounding the nucleosome free region (NFR) in a ‘peak-valley-peak’ pattern that is consistent with traditional enhancer signatures [55] (Fig. 6d).

**Fig. 7:**
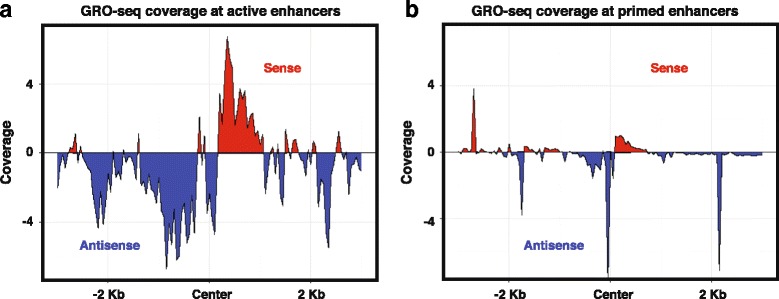
GRO-seq coverage at active versus primed enhancers. Metagene plots of GRO-seq coverage −/+ 3 Kb from the center of (**a**) active and (**b**) primed enhancers in HCT116 cells. *Red* indicates sense transcripts and *blue* indicates antisense transcripts (eRNAs)

Furthermore, while both active and primed enhancers contained comparable levels of H3K4me1, active enhancers contained larger H3K27ac levels (average coverage: 0.72 versus 0.099), and stronger eRNA sense (6 versus 1) and anti-sense (5 versus 1) read coverage compared with primed enhancers, consistent with increased enhancer activity (Fig. 7a and b). Moreover, as expected, active enhancers contain lower levels of the active promoter signature (H3K4me3) compared with the associated gene pair (average coverage: 0.247 and 1.712, respectively).

Using CIPHER in combination with our previous stringent cut-off, we also predicted enhancers in other cell lines: 38,045 active and 10,600 primed enhancers in HeLa (Fig. 6e and f), and 38,551 active and 2292 primed enhancers in K562 cells (data not shown). Collectively, these results demonstrate that our enhancer-recognition model can reliably detect enhancer elements using ChIP-seq and DNase-seq datasets in a broad range of cell lines.

## Conclusions

CIPHER is a robust, and comprehensive NGS data analysis workflow suite with numerous functions and quality control metrics. It integrates pipelines for several of the most commonly generated datasets used in current genome biology studies and features an “analysis” mode that conducts complex bioinformatics challenges such as enhancer identification and integrative dataset analysis functions. CIPHER is extremely easy to run and makes use of Docker containers so there are no dependency issues. Entire datasets can be reproduced among researchers starting from raw data in a single command. Here we re-created the results of two published studies, briefly compared CIPHER’s ease of use and speed to two other automated pipelines and provided performance metrics for several adapter decontamination and mapping tools. We further validate CIPHER’s enhancer-prediction model in various human cell lines.

Although CIPHER has combined several comprehensive and thorough pipelines for commonly used NGS approaches, there are still quite a few challenges that remain to be addressed. CIPHER’s current RNA-seq pipeline is largely optimized for typical two-type experimental designs (e.g. WT versus KO) and must be rewritten to ensure multi-design experiment DEG analysis and time-series analysis. We also plan to include pipelines for genome-wide association studies (GWAS) and de novo transcriptome assembly in the near future. Additionally, CIPHER currently only runs entire workflows, but we are aware that individuals may prefer to use only a subset of tools to complete certain tasks, thus it will be beneficial to allow this type of modular tool selection in the near future. As new or improved methods/software become available, the modular design of CIPHER will enable their smooth integration into our existing pipelines.

## Additional file

Additional file 1: Table S1. [List of KAP1-7SK snRNP target genes identified by CIPHER] (TXT 489 kb)

## Abbreviations

BAM: Binary alignment/mapping
BWA: Burrows-wheeler aligner
ChIP-seq: Chromatin immunoprecipitation followed by deep sequencing
CPU: Central processing unit
hg19: Human genome build 19
hg38: Human genome build 38
Indel: Insertion/deletion
MAQ: Mapping and assembly with qualities
NGS: Next-generation sequencing
RAM: Random access memory
RNA-seq: RNA sequencing
SNP: Single nucleotide polymorphism
